# Biogeographical Origin and Speciation of the *Anthocoris nemorum* Group

**DOI:** 10.1673/031.012.11501

**Published:** 2012-10-08

**Authors:** Min Li, Qiang Liu, Yunling Ke, Ying Tian, Gengping Zhu, Qiang Xie, Wenjun Bu

**Affiliations:** ^1^ Tianjin Key Laboratory of Animal and Plant Resistance, Tianjin Normal University. 300387, Tianjin, China; ^2^ Insect Molecular Systematic Lab, Institute of Entomology, College of Life Science, Nankai University, 300071, Tianjin, China; ^3^ Guangdong Entomological Institute, 510260, Guangzhou, China; ^4^ Patent Examination Cooperation Center, State Intellectual Property Office of the People's Republic of China, 100193, Beijing, China

**Keywords:** I6S rDNA, Anthocoridae, biogeography, *COI*, molecular clock, phylogenetics

## Abstract

The *Anthocoris nemorum* group belongs to the Anthocoridae (Hemiptera), and is an important group of predators of agricultural pests. A phylogeny was constructed in conjunction with dispersal-vicariance analysis of the *Anthocoris nemorum* group species in order to discern the relationships between the phylogeographical structuring of *A. nemorum* group species, and the effects of the Qinghai-Tibet plateau uplift. The divergence times were estimated using Bayesian inference as implemented in BEAST. A portion of the mitochondrial *COI* gene (1406 bp) and 16S rDNA (932 bp) were chosen as molecular markers to reconstruct evolutionary relationships among 10 species in this group. The combined approach, based on phylogeny, estimation of node dates, and dispersal-vicariance analyses, indicated that the phylogeographical structuring of *A. nemorum* has been primarily shaped by the two main periods of the Qinghai-Tibet plateau uplift. The DIVA optimal reconstructions suggest that *A. nemorum* diversified from the Miocene to the Pleistocene from a center of origin in the Hengduan Mountains. The rapid uplift of Mountain ranges associated with the uplift of the entire Qinghai-Tibet plateau may have promoted rapid divergence in the *A. nemorum* group. Vicariance and dispersal were both essential in shaping the present distribution patterns of *A. nemorum*.

## Introduction

The Qinghai-Tibet plateau is known as “the roof of the world,” with an average elevation of approximately 4500 m (Zheng 1996). This area had been listed as one of the world's biodiversity hotspots (Wilson 1992; Myers et al. 2000), and is a model region for biodiversity research (Jin et al. 2008). On the plateau, the Himalayas are located in the south, the Kunlun Mountains and the Qilian Mountains in the north (Yin and Harrison 2000), and the Hengduan Mountains in the east. The Qinghai-Tibet plateau began to gradually uplift after the India-Asia collision (Le Fort 1996; Zhang et al. 2008), and is still in the process of uplifting (Huang et al. 2009). The current elevation of the Qinghai-Tibet Plateau was assumed to be not reached until as recently as 4 million years (myr) ago (Sun & Zheng 1998). The extremely complex topography was formed during the uplift, and the significant increase in geological and ecological diversity that accompanied such an uplift promoted rapid divergence and speciation in small and isolated populations (Liu et al. 2006), which has been assumed to be one of the reasons for high diversity in this region (Axelrod et al. 1996). However, because of the complicated topography and limited access, this region is among the areas where biodiversity studies have been limited (Hewitt 2007).

The species of the *Anthocoris nemorum* group will be of interest for the biogeography of other species' range and speciation scenarios in this region. The genus *Anthocoris* Fallén, 1814, which includes about seventy species, is the second largest genus in the family Anthocoridae (Heteroptera) (Gross 1954; Henry and Froeschner 1988; Péricart 1996; Carpintero 2002). Hill (1957) divided the six North American species into three groups according to body form, color, and male genitalia. Péricart (1972) divided the west Palaearctic species of *Anthocoris* into six groups, based on characters of the forewing, sternum II of the abdomen, and male genitalia. Bu and Zheng (2001) separated the Chinese *Anthocoris* into nine groups, according to body shape, structure of the pronotum, color and shining pattern of the forewing, and structures of the second and third abdominal sterna and paramere. Ke and Bu (2007) divided the world species of *Anthocoris* into thirteen groups mainly based on the copulatory tubes of females.

The *Anthocoris nemorum* group, one of the thirteen groups in the genus *Anthocoris*, includes eleven species: *A. alpinus* Zheng 1984, *A. expansus* Bu 1995, *A. kerzhneri* Bu and Zheng 2001, *A. limbatus* Fieber 1836, *A. montanus* Zheng 1984, *A. nemorum* Linnaeus 1761, *A. pricart* Bu and Zheng 2001, *A. qinlingensis* Bu and Zheng 1990, *A. zoui* Bu and Zheng 2001, *A. antevolens* White 1879 and *A. musculus* Say 1832. These eleven species share the following morphological characters: the hemelytron is shining, with short, decumbent pubescence; the basal half or the whole endocorium is light-colored; the abdominal sternum II or III does not have a membranous area, the paramere is lamellate, and the outer portion is expanded outward forming a longitudinal groove. The copulatory tube is of the *A. nemorum* or *A. alpinus* type (Ke and Bu 2007).

**Figure 1.**
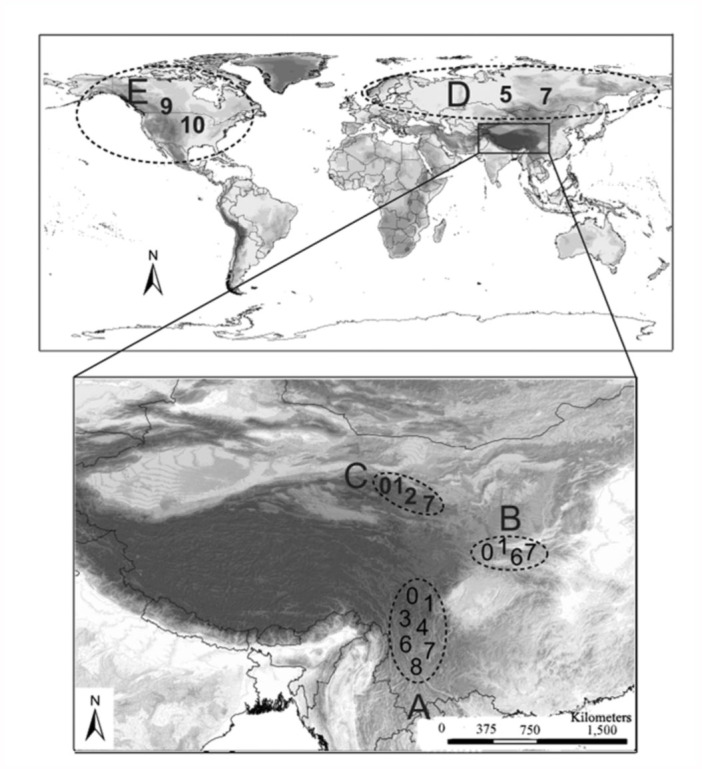
The distribution of the *Anthocoris nemorum* group. 0: *A. montanus*; 1: *A. expansus*; 2: *A. pericarti*; 3: *A. zoui*; 4: *A. alpinus*; 5: *A. limbatus*; 6: *A. qinlingensis*; 7: *A. nemorum*; 8: *A. kerzhneri*; 9: *A. antevolens*; 10: *A. musculus*. Areas of distribution of the *A. nemorum* group as defined in this study: Hengduan Mountain region (A); Qinling Mountain area (B); Qilian Mountain area (C); The northern part of the Palaearctic region (D); Nearctic region (E). High quality figures are available online.

Two species of *A. nemorum* are widespread in the Palaearctic region, and two are distributed in the Nearctic areas (Ke and Bu 2007). Most of the species are endemic to a certain altitude (2000–3000 m) along mountain ranges at the edge of the Qinghai-Tibet plateau (Figure 1). Geological, ecological, and biogeographic histories have played important roles in shaping the current species distribution pattern (Willis and Whittaker 2002; Qian et al. 2005). The distribution pattern of *A. nemorum* could be corrected by associating the physiognomy, ecology, and formation histories of the Qinghai-Tibet plateau in southwest China.

Our work is the first attempt to apply molecular evidence in reconstructing the evolutionary relationships of species within the *A. nemorum* group. We also reconstructed the ancestral distributional areas, dispersal route, and divergence times for *A. nemorum*, and we discuss speciation scenarios in the context of biogeography.

## Materials and Methods

### Taxa Sampling

A total of 13 species were included in these analyses, and one individual per species was used. Ten species of the *A. nemorum* group were in-groups, except for one rarely-distributed species, *A. musculus*. Three outgroup species were selected: *A. miyamotoi* Hiura 1959, *Temnostethus reduvinus* (Herrich-Schaeffer 1853), and *Orius sauteri* (Poppius 1909). *A. miyamotoi* Hiura 1959, is a congeneric species with the *A. nemorum* group. *A. antevolens* is similar to *A. musculus* in morphological characters and distribution pattern, so *A. antevolens* was used as a representative of both species for the analysis in this study. The genus *Temnostthus* also belongs to the tribe of Anthocorini, and is closely related to *Anthocoris*. There is a fossil species, *Temnostethus blandus* Statz and Wangner 1950, from the Oligocene of Germany in the same genus as extant species, which could be used as a calibration point to calculate the divergence time of the genus (see section “Estimation of divergence times”). *Orius sauteri* (Poppius 1909), belongs to the tribe of Oriini Carayon 1958, which is a sister tribe to Anthocorini (Ke 2004).

The samples were directly identified by taxonomic experts of Anthocoridae Wenjun Bu and Yunling Ke. The voucher specimens are deposited in the Institute of Entomology, College of Life Sciences, Nankai University, Tianjin, China. The details of the taxa information are shown in Table 1.

### Laboratory procedure

Genomic DNA extraction followed a modified cetyltrime thylammonium bromide (CTAB-based extraction protocol) method (Doyle and Doyle 1987; Cullings 1992). The DNA fragments were amplified by polymerase chain reaction. The *COI* sequence was amplified using primer pairs LCO1490 (5′GGT CAA CAA ATC ATA AAG ATA TTG G-3′)(Folmer et al. 1994)/TL2-N-3014 (5′TCC AAT GCA CTA ATC TGC CAT ATT AT -3′) (Simon et al. 1994). We designed a pair of primers to amplify 16S rDNA, 16S1(5′ - GTA AAA TTC TAC AGG GTC TTC TCG TCT A - 3′) and 16S-2 (5′ — AGG TGA GAT AAG TCG TAA CAA AGT A - 3′) based on the available information on relevant sequences in GenBank.

Reactions were performed in 25 uL reaction buffer (10 × PCR buffer, 15 mM MgCl_2_, 200 µM dNTPs, 1 µM of each primer, 50 ng template DNA and 1 unit Taq DNA polymerase). Temperature cycling was carried out in a Biometra T Gradient Cycler with annealing temperatures of 50° C (*COI*) and 55° C (16S rDNA) for 35 cycles. The polymerase chain reaction products were separated in a 1.0% low-melting-temperature agarose gel and purified by Agarose Gel DNA Purification Kit (Takara, www.takara-bio.com). Purified polymerase chain reaction products were directly sequenced by the SunBio Company (www.sunbio.com) with the same primers used for polymerase chain reaction.

### Sequence alignment and phylogenetic analyses

Sequence alignment was performed using the software program Clustal X (Thompson et al. 1997) with default parameters. The nucleotide substitutions and Pairwised distance (based on the Kimura-2-parameter) of each gene were computed using MEGA version 4.0 (Tamura et al. 2007). Phylogenetic analyses were done by Maximum Parsimony (Swofford and Begle 1993) carried out in PAUP 4.0b10 (Swofford 2003), and the Bayesian (Yang and Rannala 1997) carried out in MrBayes 3.1.2 (Huelsenbeck and Ronquist 2001) based on combination data matrices. Heuristic searches using the TBR algorithm were performed to produce unweighted parsimony analyses of various datasets. Bootstrap values were generated in PAUP from 1000 replicates, each with ten random-addition heuristic searches. Best-fitting nucleotide substitution models for each gene were selected by Modeltest 3.7 (Posada and Crandall 1998) using the Akaike Information Criterion (AIC). GTR+I+G model parameters were selected for the *COI*, and GTR+I model for 16S rDNA. In the Bayesian analyses, variation was partitioned among genes.Gene-specific models were used, and all parameters were free to vary independently within each partition. The ‘unlink’ command was used in MrBayes, so that each gene was allowed to evolve at a different rate. Two independent runs with 3,000,000 generations were implemented in parallel, and sampling frequency of every 100 generations was employed. When the average deviation of split frequencies fell well below 0.01, the two runs were stopped. For each running, the first 750,000 generations were discarded as burnin, and the remaining trees were used to construct a 50% majority-rule consensus tree.

### Estimation of divergence times

Divergence times were estimated using Bayesian inference as implemented in BEAST version 1.4.7 (Drummond and Rambaut 2007). Divergence times were calculated as 95% highest posterior density intervals on a time-measured phylogeny. Substitution rates for the two genes and the three codon positions were unlinked in order to be estimated independently, and each gene was allowed to evolve at a different rate. The GTR+I+G substitution model was used for the *COI* gene, and the GTR+I model was selected for the 16S rDNA gene. The ‘relaxed clock’ rate variation model was used with lognormal distribution of rates and Yule tree priors, which are appropriate for species-level phylogenies (Drummond et al. 2006, 2007). The final analysis consisted of two independent Markov chain Monte Carlo analyses; each chain was run for 30,000,000 generations with parameters sampled every 1000 steps. Independent runs converged on very similar posterior estimates and were combined using LogCombiner version 1.4 (Drummond and Rambaut 2006). Tracer 1.2 (Drummond and Rambaut 2006) was used to confirm adequate mixing of the Markov chain Monte Carlo chain, appropriate burn-in (25%), and acceptable effective sample sizes (> 200). The logged trees were summarized using TreeAnnotator 1.4.7, and displayed in FigTree 1.1.2.

One fossil record as an external calibration was used to date the divergence times within the *A. nemorum* group. *Temnostethus blandus* Statz & Wagner 1950, a fossil species, was assigned to the Oligocene, 23–35 myr ago. The minimum age of the fossil was used to place a minimum bound on the age of the respective nodes (Benton and Donoghue 2007; Donoghue and Benton 2007; Ho 2007). It assumes that the actual divergence is more likely to have occurred earlier than the fossil (Ho 2007). Therefore, we used 23 myr ago as the calibration point to calibrate the node splitting of *Temnostethus* and *Anthocoris*.

### Biogeographic analyses

We reconstructed ancestral areas by means of dispersal-vicariance analysis using DIVA 1.1 (Ronquist 1996, 1997), which has been commonly used for reconstructing ancestral areas in biogeographic studies (Xiang et al. 2005, 2006; Lang et al. 2007; Jeandroz et al. 2008). The tree topology resulting from the BEAST analyses was used as the framework for the reconstruction of optimal ancestral distribution in DIVA 1.1. Areas selected in this study were based on the geographic distribution of the species. Four areas were considered in the analysis (Figure 1). The Hengduan Mountains area (A) includes western and northwestern Yunnan, western Sichuan, and the southeastern Qinghai-Tibet plateau. The Qinling Mountains (B) area includes the area from the Gansu-Qinghai border in the west through Shaanxi to central Henan in the east. The Qilian Mountains (C) area includes the southern part of Gansu and the northeast part of the Qinghai-Tibet plateau. The northern part of the Palaearctic (D) includes the area north of the Qilian Mountains and Qinling Mountains in the Palaearctic. The Nearctic region (E) is endemic to a certain altitude (2000–3000 m) along mountain ranges at the edge of the Qinghai-Tibet plateau (Figure 1). As the range of most taxa are mainly constrained to three areas (A, B and C; Figure 1), the ancestors are assumed to not have had a higher ability to disperse (and maintain genetic cohesion across the species range) than the descendants.

To better search for ancestral distribution, the optimized command “maxareas” was used in order to impose a constraint on the number of unit areas allowed in ancestral distributions. The maximum number of ancestral areas was constrained to three (maxareas = 3).

## Results

### Data Characteristics

Nucleotide sequence alignment resulted in a 16S rDNA data partition of 932 characters, a *COI* data partition of 1406 characters, and a total combined data set of 2338 characters. For the *COI* data partition, 580 sites (41.3%) were variable, and 434 sites (30.9%) were parsimony informative. The proportions of variable sites and informative characters were higher in 16S rDNA, which yielded 468 sites (50.1%) and 374 sites (40%) respectively. The ratios of the number of transitions to the number of transversions for these two genes were 0.4 (16S rDNA) and 0.9 (*COI*). 16S rDNA (A: 47.7%, T: 33.9%) was AT rich in nucleotide composition, which is common in insect mitochondrial DNA sequences (Crozier and Crozier 1993). There were 1052 variable sites (44.9%), and 814 informative characters (34.8%), in the combined data set of these two genes, and the nucleotide composition was A: 39.2%, T: 34%, G: 12.2%, C: 14.7%. The average ratio of transitions to transversions was 0.6. The two-pore domain potassium channel distances of two genes (*COI* and 16S rDNA) are shown in the Table 2.

**Figure 2.**
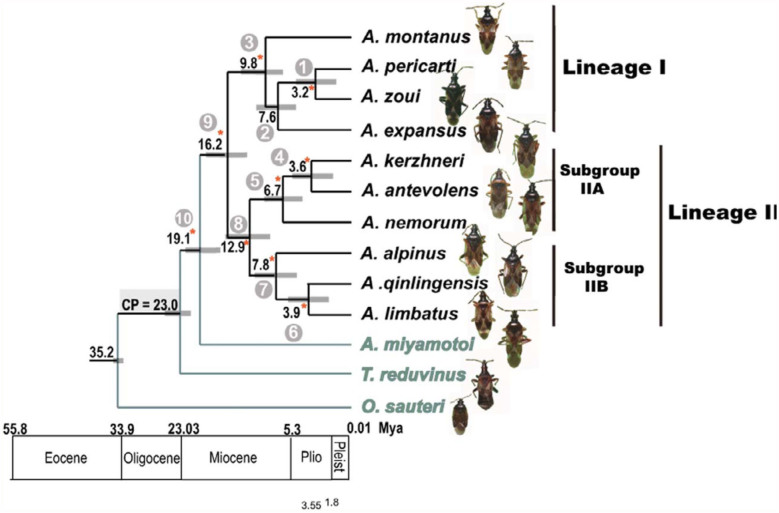
Phylogentic trees using different analytical methods (MP and Bayesian) yielded similar results. Ten nodes are shown in the figure, and the details supporting the value of each node based on the two methods are shown in Table 3. Chronogram *of Anthocoris nemorum* group showing estimates of divergence times obtained with BEAST. Numbers at nodes are the average estimates of times in myr ago. Grey bars are 95% highest posterior density intervals. The calibration point was set to 23.0 myr ago according to the fossil record of *Temnostethus*. Asterisks indicate nodes with both posterior probability > 90%. High quality figures are available online.

### Divergence Times

The BEAST Markov chain Monte Carlo runs yielded high effective sample sizes (> 500) for all relevant parameters (i.e., branch lengths, topology, and clade posteriors), indicating adequate sampling of the posterior distribution. The maximum credibility tree (Figure 2) retrieved from the combined analyses (TreeAnnotator version 1.4; Drummond and Rambaut 2006) is identical to the MrBayes consensus tree in topology and posterior support values. A chronogram representing the divergence times of the principal lineages of the *A. nemorum* group is shown in Figure 2.

### Phylogenetic and Biogeographic Analyses

The combined 16S-*COI* data sets produced completely resolved trees with similar topologies, irrespective of the different analytical methods employed. The single maximum parsimony tree produced by the parsimony method was similar with Bayesian and the maximum credibility tree from BEAST. The resolved trees showed two main lineages, designated as lineage I and II. The latter was further divided into IIA and IIB subgroups (Figure 2). The details supporting the value of each node based on the two methods are shown in Table 3.

**Figure 3.**
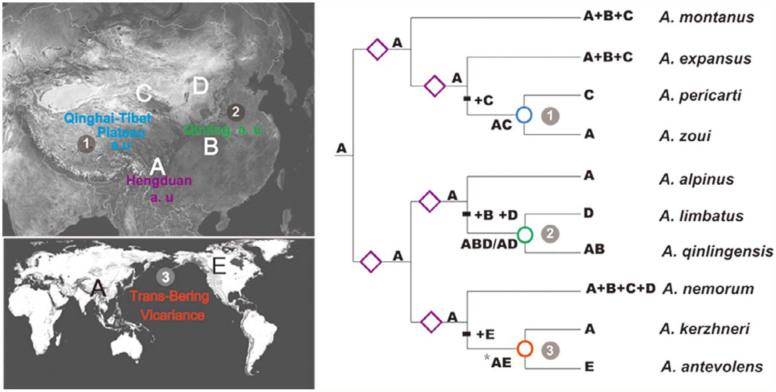
Summary of the optimal reconstructions of ancestral distributions of the *Anthocoris nemorum* group using dispersalvicariance analysis (DIVA). At each node, the optimal distribution is given; alternative, equally optimal distributions are separated with slash marks. Symbols: circle = vicariance event; rhomb = duplication (sympatric speciation) event; plus (+) = dispersal event. One unambiguous event is indicated in the reconstruction (*): Nodes where extinction events were inferred because the subsequent vicariance event takes place between areas that are not geographically adjacent (Figure 1). Abbreviations: a.u., acute uplift events. High quality figures are available online.

Lineage I contains four species: *A. montanus, A. zoui, A. pericarti*, and *A. expansus* (Figure 2). *A. pericarti* has a close relationship with *A. zoui* by a well-supported (node 1, 90 MP, 1.00 Baysian; Table 3.). *A. expansus* is the sister group of *A. zoui* and *A. pericarti. A. montanus* is placed as a basal group to lineage I. Lineage I is supported with significant support value (node 3, 100 MP, 1.00 Baysian; Table 3). In Lineage II, *A. nemorum* is placed as a basal group to the subgroup IIA in Bayesian with a significant support value (node 5, 1.00 Baysian; Table 3; Figure 2). In the MP method, *A. nemorum* is the sister species of *A. kerzhneri*, and this is the only different topology between the two methods. However, this node based on MP is probably less reliable because it lacks strong bootstrap support (BP = 47 not shown). Subgroup IIB includes *A. alpinus, A. qinlingensis*, and *A. limbatus* (Figure 2). *A. qinlingensis* and *A. Iimbatus* are more closely related to each other than to *A. alpinus*. This subgroup is also supported with significant support value (node 7, 100 MP, 1.00 Baysian; Table 3).

A DIVA constrained exact search limiting ancestral distributions to no more than three areas resulted in two alternative, equally optimal reconstructions, each requiring 10 dispersals between the areas. The optimal area reconstructions at each ancestral node are summarized in Figure 3. All reconstructions postulate the existence of a restricted ancestor of the *A. nemorum* group distributed in the Hengduan Mountatins (A). Therefore, the presence of the *A. nemorum* group in areas such as the Qingling Mountains (B), Qilian Mountains (C), and the northern part of the Palaearctic (D) and Nearctic region (E) is considered to be the result of subsequent dispersals from the ancestral distribution.

## Discussion

The Qinghai-Tibet plateau began a gradual uplift after the India-Asia collision (approximately 50–40 myr ago) (Le Fort 1996; Zhang et al. 2008). The more recent abrupt uplift has been divided into two phases, A and B. During stage A, the initial major uplift of the Qinghai-Tibet plateau has been dated from the Early Miocene, 23-15 to 8 myr ago (Patriat and Achache 1984; Klootwijk et al. 1985). In stage B, acute uplift proceeded, and the altitude of the Qinghai-Tibet plateau went from 3000 m to an average of 5000 m during the past 4 million years (Cui et al. 1996; Shi et al. 1998; Zhang et al. 2000; Zheng et al. 2000; An et al. 2001). The combined approach based on phylogeny, estimation of node dates, and dispersalvicariance analyses, indicated that the phylogeographical structuring of *A. nemorum* group species has been primarily shaped by the two main periods of the Qinghai-Tibet plateau uplift. For the basal nodes forming the backbone of the tree (node 3, 5, 7, 8, 9 and 10; Figure 2.) of the *A. nemorum* group, the age of the divergence was estimated at about 19.1-6.7 myr ago, coinciding with the first period of uplift of the Qinghai-Tibet plateau (Stage A). The age of the most recent common ancestors (node 1, 4, 6) was estimated at about 3.9–3.2 myr ago. which falls within the temporal framework of the second stage B uplift. Thus the divergence dates and the phylogeographical structuring (Figure 3) suggest that the uplift of the Tibetan Plateau played a fundamental role in the diversification of the *A. nemorum* group.

The DIVA ancestral area reconstructions postulated that the ancestor of the *A. nemorum* group was originally present in the Hengduan Mountains region (A), where it underwent duplication (speciation within the area), and gave rise to two different lineages: lineage I and lineage II (Figure 3). The Hengduan Mountains are located on the southeastern edge of the Qinghai-Tibet plateau (Wu 1988), comprising a series of spectacular north-south ridges, alternating with deep valleys. These steep mountains and valleys might provide novel ecological opportunities for accelerated speciation in this region (Gao et al. 2007; Yue et al. 2009). According to previous studies, the Hengduan Mountains are not only a center of biodiversity, but also a center of active speciation (Deng et al. 2009; Huang et al. 2009). The acute rising of the Hengduan Mountain with the entire uplift of the Qinghai-Tibet plateau in stage A (Jin et al. 2003) might have introduced the ecological opportunity necessary for *A. nemorum* group speciation in this region. The proposed biogeographic scenarios and the divergence-time estimated by BEAST also suggested that the *A. nemorum* group originated from the Hengduan Mountains, and diversified by several rapid speciation events beginning in the Miocene.

The ancestor of the species of Lineage I remained in the Hengduan Mountains (A), underwent duplication events, and afterward dispersed to the Qilian Mountains (C) (Figure 3). One vicariance event (A/C, node 1; Figure 3) separated *A. zoui* in the Hengduan region (A) from *A. pericarti* in the Qilian area (C). The mean age of the divergences estimated by BEAST was 3.2 myr ago. (1.3–5.1 myr ago) (node 1; Figure 2), within the Pliocene-Pleistocene period. The acute uplift of the Qinghai-Tibet plateau in this period may be an explanation for the divergence of these two species.

The Qinghai-Tibet plateau began its intensive uplift in the Pliocene-Pleistocene, and the altitude rose from 3,000 m to its present height (Cui et al. 1996; Shi et al. 1998; Zhang et al. 2000; Zheng et al. 2000; An et al. 2001). Since *A. nemorum* group species are mainly restricted to ranges from 2000 to 3000 m in elevation (Bu and Zheng 2001), the Qinghai-Tibet plateau could have been a proper route for the *A. nemorum* group species to disperse before the early Pleistocene. However, starting in the early Pleistocene, the dispersal of *A. nemorum* group species was stopped by the acute uplift phase of the Qinghai-Tibet plateau. This could be the explanation for the vicariance divergence from *A. pericarti* in the Qilian Mountains (C), and *A. zoui* in the Hengduan Mountains (A) (node Figure 3.).

The ancestor of the species in lineage II in the Hengduan Mountains region (A) also underwent duplication events, and gave rise to two different subgroups (Figure 3). There had been several dispersal events from the Hengduan Mountains (A) to the other areas (+B, +D, +E; Figure 3) in lineage II.

Two dispersal events occurred at internal branches that were later split by vicariance (allopatric speciation) events (node 2, 3; Figure 3). First, the ancestor of the species in subgroup IIA originated from the Hengduan Mountains (A), experiencing a dispersal event from A to the Nearctic area (E), and later underwent a vicariance event (A/E, node 3; Figure 3). There were two different routes between Asia and North America: the Bering land bridge, and the North Atlantic European—North American land bridge. The North American land bridge persisted until at least the early Eocene (50 myr ago) and the Bering land bridge was available several times during the Tertiary, Miocene, and Pleistocene glaciations (Wen 2000; Sanmartin et al. 2001; Milne 2006). During the Pliocene to Pleistocene period, climatic, geographic, and vegetational changes presumably resulted in massive trans-Beringian vicariance, and the division was reinforced around 3.5 myr ago during the Late Pliocene (Sanmartin et al. 2001). The corresponding age of the separation of *A. kerzhneri* from *A. antevolens* is estimated to be 3.6 myr ago (1.7–5.5 myr ago) (node 4; Figure 2) by BEAST, coinciding with the trans-Beringian vicariance, which may have formed the differentiation observed today (Figure 3). Also, due to the distribution of *A. antevolens* in the northern and western part of North America (Horton et al. 2007), we postulate that the ancestor of *A. antevolens* and *A. kerzhneri* migrated from East Asia to North America via the Bering land bridge, and later underwent a vicariance event with the disappearance of the Bering land bridge in Pliocene to Pleistocene. The present disjunct distribution of *A. kerzhneri* in the Hengduan Mountain regions (A) and *A. antevolens* in the Nearctic area (E) (Figure 3) is probably the result of the extinction or unknown occurrences of this clade in the intermediate areas. This extinction may have been caused by the Quaternary glaciation. The Hengduan Mountains have been suggested as a potential refugium during the Quaternary glaciation (Chen et al. 2008a, b; Feng et al. 2009). Nevertheless, more work is needed to put forward robust hypotheses.

*A. antevolens* was suspected to be a complex of species in the Nearctic region in one recent study (Horton et al. 2007), which means that *A. antevolens* may still be undergoing divergence in the Nearctic area. For more accurate dating, more specimens sampling is needed in these areas.

The biogeographical reconstruction (Figure 3) indicated that the ancestor of the species of the subgroup IIB experienced a dispersal event from the Hengduan Mountains (A) to the Qinling Mountains (B) (since this species is only distributed in the southern area of the Qingling Mountains) and the northern part of the Palaearctic (D), after undergoing a vicariance event (AB/D, node 2; Figure 3) between *A. limbatus* in the northern part of the Palaearctic (D) region and the sister species of *A. qinlingensis* in the Hengduan Mountains (A) and Qinling region (B). The other equally optimal reconstruction indicated that the ancestor dispersed from the Hengduan Mountains (A) to the Palaearctic (D) region, undergoing a vicariance event (A/D), and afterward undergoing a recent dispersal event to the Qinling Mountains (B).

The mean age of the divergences estimated by BEAST was 3.9 myr ago (1.8–6.0 myr ago) (node 6; Figure 2). The acute Qinling uplift in this period may be an explanation for the evolutionary divergences.

The Qinling Mountains, extending about 1,500 kilometers across central China from the Gansu-Qinghai border in the west through Shaanxi to central Henan in the east, form a natural dividing line between China's subtropical and warm-temperate zones (Wu 1979). It is also the important natural geographic dividing line between South and North China (Chen and Cao 1986; Zhang and Chen 1997). The Qinling Mountains began an acute uplift in Pliocene—Pleistocene period (Xue and Zhang 1996). This could explain the vicariance divergence from *A. qinlingensis* in the Hengduan Mountains (A) and the Qingling Mountains area (B), and *A. limbatus* in the northern part of the Palaearctic region (D), and the equally optimal reconstruction event A/D. This explanation agrees with other biogeographical studies that consider Qinling responsible for evolutionary divergences between South and North China lineages (Wang et al. 1997; Bai et al. 2005; Zhao et al. 2008).

The molecular clock for arthropod mtDNA suggested by Brower (1994) indicated that the rate of pairwise divergence was approximately 2.3% sequence divergence per million years. In the *A. nemorum* group, the rate of pairwise divergence was approximately 0.6–1.3% (16S rDNA) and 1.2– 1.8% (*COI*) sequence divergence per million years based on the Pairwised distance (Table 2) and the divergence times (Figure 2).This result agrees with other studies in Heteroptera that consider the standard 2.3% sequence divergence per million years may be an overestimate of divergence rates in Heteroptera (Andersen et al. 2000; Damgaard and Zettel 2003; Pfeiler et al. 2006).

## Scenario for the two widespread species in the Palaearctic region

Habitat specificity can be a restriction factor in species distributions (Miller and Duncan 2003). The altitude restriction (2000–3000 m) of the *A. nemorum* group species may be one reason why most of them are endemic to that altitude along different mountain ranges at the edge of the Qinghai-Tibet plateau. In the dispersal process, most of the species are temporary visitors, and do not breed in lower or higher environments. However, a few species in the *A. nemorum* group can breed in relatively lower or higher altitudes in the dispersal process. One species, *A. limbatus*, has become a true resident of the relatively low altitude of 1000–1500 m, which is widespread in the northern part of the Palaearctic region. Another species, *A. nemorum*, can accommodate both the higher and lower altitudes, and became the widespread species in the Palaearctic region.

**Table 1.**
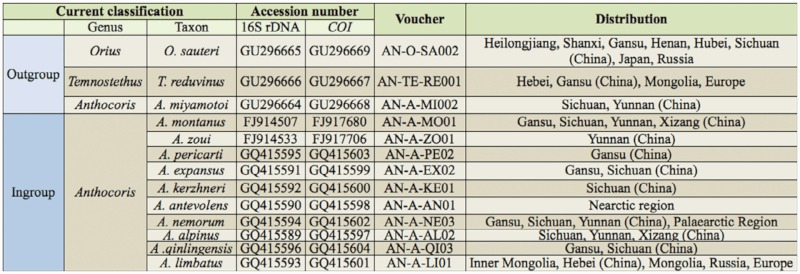
Taxa used in this study.

**Table 2.**
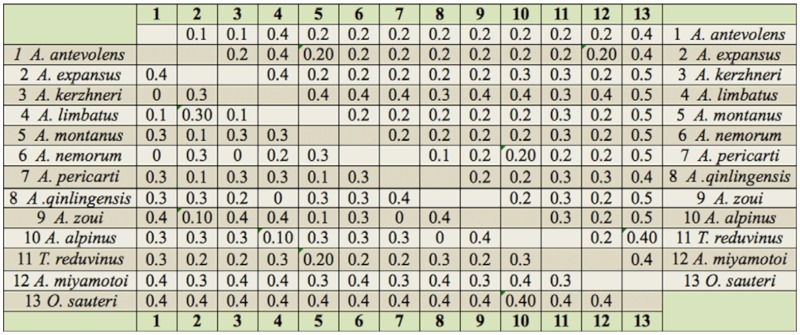
The Kimura two-parameter distances of the 16S rDNA gene (lower-left)/ COI (upper-right) between species of the *Anthocoris nemorum* group.

**Table 3.**
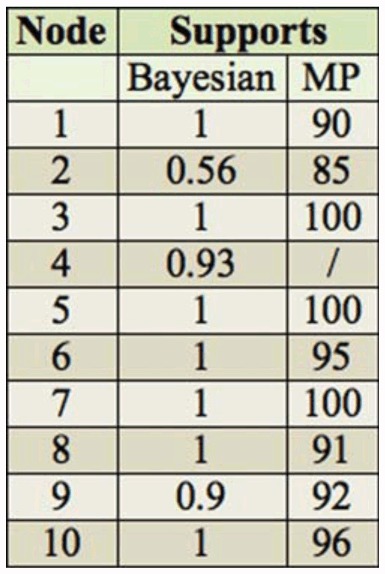
The supports values of each node based on the two methods (Node label see Figure 2).
